# Targeted Nanobody-Based Molecular Tracers for Nuclear Imaging and Image-Guided Surgery

**DOI:** 10.3390/antib8010012

**Published:** 2019-01-11

**Authors:** Pieterjan Debie, Nick Devoogdt, Sophie Hernot

**Affiliations:** Laboratory for in vivo Cellular and Molecular Imaging, ICMI-BEFY/MIMA, Vrije Universiteit Brussel, Laarbeeklaan 103, 1090 Brussels, Belgium; pieterjan.debie@vub.be (P.D.); ndevoogd@vub.be (N.D.)

**Keywords:** single-domain antibody fragments, molecular imaging, molecular therapy, nuclear imaging, targeted fluorescence imaging, intraoperative imaging

## Abstract

Molecular imaging is paving the way towards noninvasive detection, staging, and treatment follow-up of diseases such as cancer and inflammation-related conditions. Monoclonal antibodies have long been one of the staples of molecular imaging tracer design, although their long blood circulation and high nonspecific background limits their applicability. Nanobodies, unique antibody-binding fragments derived from camelid heavy-chain antibodies, have excellent properties for molecular imaging as they are able to specifically find their target early after injection, with little to no nonspecific background. Nanobody-based tracers using either nuclear or fluorescent labels have been heavily investigated preclinically and are currently making their way into the clinic. In this review, we will discuss different important factors in nanobody-tracer design, as well as the current state of the art regarding their application for nuclear and fluorescent imaging purposes. Furthermore, we will discuss how nanobodies can also be exploited for molecular therapy applications such as targeted radionuclide therapy and photodynamic therapy.

## 1. Introduction

Molecular imaging has been defined as “the visualization, characterization, and measurement of biological processes at the molecular and cellular level in living subjects” [1]. The technique makes use of molecular tracers in probing for biomarkers expressed in (patho)physiological processes. Molecular tracers most often consist of a targeting moiety to direct the tracer and a signaling moiety for detection. Following administration, the molecular tracer will specifically accumulate in areas where the biomarker reveals itself, while unbound tracer will be eliminated. The bound molecular tracer is then visualized by means of an appropriate imaging modality. Radioactivity and fluorescence are particularly suited for the application because of their high detection sensitivity. Yet, the choice of detection system and associated signaling molecule is directly linked to the intended application. In the clinic, nuclear molecular imaging is mainly used as a noninvasive technique for diagnosis of diseases, treatment follow-up, or early patient stratification according to the expression of predictive biomarkers [2]. Contrarily, as fluorescence signals have limited depth penetration (several mm), the use of fluorescence imaging is restricted to imaging of the skin surface and interventional procedures, i.e., surgery, endoscopic, and intravascular imaging. Over the last decade, fluorescence-guided interventions have gained interest as a method to assist surgeons in real-time by demarcating cancerous tissues for precise and complete resection [3], by highlighting healthy tissues that should be preserved [4,5], by guiding biopsy [6,7], or by interrogating suspicious lesions in vivo at the molecular level [8].

The design of molecular tracers is driven by certain requirements: the tracer should remain stable in vivo, it must accumulate specifically and in sufficient amounts into the tissue of interest, no or low uptake in nontargeted tissues and organs should be detected in order to reach high contrast, and an appropriately fast imaging time point and sufficiently extended imaging window should be attained. These parameters are predominantly defined by the pharmacokinetics of the tracer, and thus by the choice of targeting agent. Several classes of targeting agents can be considered, including antibodies, antibody fragments, scaffolds, peptides, or small molecules [9]. Antibodies, given their natural capacity to recognize specific epitopes as part of the body’s humoral immune system, have been extensively considered for in vivo targeting. However, their large size results in slow systemic clearance and hampers deep tissue penetration [10]. In consequence, molecular imaging performed using antibody tracers often yields low-contrast images with low specificity, and only several days after tracer administration [11]. This review will focus on nanobodies as a platform technology, since nanobody-based tracers have been shown to possess unique characteristics in terms of versatility, specificity, and the short time needed to attain high contrast [12]. Following the description of what nanobodies are, we will give an overview of the application of radiolabeled nanobodies in nuclear medicine. Subsequently, we will discuss how the attractive properties of nanobodies can also be exploited for fluorescence-guided surgery and photodynamic therapy. The different aspects that are important to their design and utilization as molecular tracers in these fields will be addressed in more detail.

## 2. Nanobodies and Their Unique Properties

Conventional antibodies are Y-shaped and are composed of two light and two heavy polypeptide chains. In camelids, besides these, a substantial fraction of IgG-like antibodies devoid of light chains also occur naturally [13]. Although only half of the complementarity-determining regions (CDRs) needed for antigen recognition are present, these heavy-chain-only antibodies retain a similar affinity and specificity to conventional IgGs. This interesting finding has led to the exploitation of the variable domain of heavy-chain-only antibodies for many biotechnological and medical applications. This monomeric domain of 12–15 kDa in size and with nanometer-range dimensions is often referred to as a single-domain antibody (sdAb) or the variable domain of a heavy-chain antibody (V_H_H), and has been given the commercial name Nanobody^TM^ [14].

**Figure 1:**
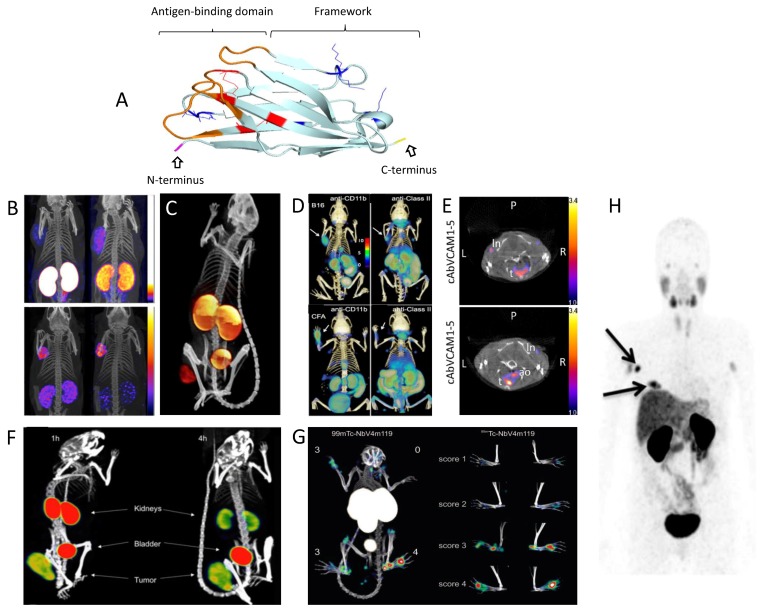
Schematic representation of the structure of a nanobody and illustrative positron emission tomography (PET) and single photon emission computed tomography (SPECT) preclinical and clinical images obtained using nanobodies that are labeled with distinct radionuclides in diverse medical applications, from oncology, immunology, atherosclerosis, and arthritis to the theranostic imaging of a radiotherapeutic probe. (**A**) Ribbon diagram of the nanobody 2Rs15d. The complementarity-determining regions (CDRs) are shown in orange, lysines (used for random conjugation methods) in blue, and cysteines and cysteine bridges in red. The C-terminus (in yellow) can be easily genetically modified for site-specific conjugation methods. (**B**) SPECT/Computed Tomography (CT) images of the biodistribution of ^111^In-labeled JV7 nanobodies at 3 h post-injection in PSMA+ tumor-bearing mice (on the left shoulder). Effect on renal retention by the removal of tags (top panels: Myc–Cys-tagged nanobody, bottom: Cys-tagged nanobody) and coinjection of positively charged amino acids and gelofusin (left panels: no injection, right panels: with coinjection) is shown. Adapted from [48]. (**C**) SPECT/CT imaging of an EGFR+ tumor-bearing mouse 1 h after injection of ^99m^Tc-labeled 7C12 nanobody. Adapted from [64]. (**D**) PET/CT immune cell imaging 90 min after injection of ^18^F-labeled nanobodies against murine CD11b and major histocompatibility complex (MHC) class II. Top: C57Bl/6 mice inoculated with B16 tumor cells on the left shoulder; bottom: animals injected with complete Freund’s adjuvant on the left paw. Adapted from [72]. (**E**) SPECT/CT coronal image taken at 2–3 h post-injection of ^99m^Tc-labeled cAbVCAM1-5 nanobody, showing uptake in atherosclerotic lesions (ao) of ApoE^-/-^ mice (bottom) and absence of signals in the aortic arch of C57Bl/6J mice (top) [43]. (**F**) SPECT/CT images of the biodistribution of the ^131^I-labeled 2Rs15d therapeutic nanobody in a mouse model with subcutaneous HER2+ xenograft at 1 and 4 h post-injection. Adapted from [54]. (**G**) SPECT/CT imaging of arthritis in a mouse model with a VSIG4/CRIg-specific ^99m^Tc-labeled nanobody. Adapted from [73]. (**H**) PET/CT image of the biodistribution of ^68^Ga-labeled anti-HER2 nanobody in a breast cancer patient 90 min post-injection showing uptake in breast tumor lesions. Adapted from [23].

Crystallographic studies of nanobodies revealed, as for conventional V_H_ fragments, a classical immunoglobulin fold with nine antiparallel β-strands forming two β-sheets, connected through a conserved disulfide bridge (Figure 1A). In camel-derived nanobodies, more so than for llama-derived nanobodies, an additional disulfide bridge is often present between the CDR1 and CDR3 or the CDR2 and CDR3 loops. The three CDR loops are located near the amino (N)-terminal end of the domain and opposite to the carboxy (C)-terminal end. However, adaptations are essential to compensate for the absence of a variable light chain (V_L_). On the one hand, four hydrophobic residues which normally interact with the V_L_ domain are changed to more hydrophilic ones [15,16,17]. This results in a structure with improved water solubility and which is less prone to aggregation [18]. On the other hand, the lack of three extra loops for antigen recognition is compensated by elongated CDR1 and CDR3 loops that are able to adopt alternative canonical structures. As a consequence of the often more convex shape of the paratope, nanobodies tend to bind epitopes located within cryptic clefts [19]. 

An important advantage of nanobodies is their high thermal and chemical stability. They typically exhibit melting temperatures above 60 °C, and antigen-binding activity is retained even after prolonged incubation at such high temperatures [20,21]. These properties open opportunities for novel chemical modifications and labeling methods. 

Despite these differences, nanobodies are considered to be very weakly immunogenic due to their high degree of homology with human variable heavy-chain (V_H_) fragments [22]. This has since been confirmed by several clinical trials where no immunogenicity or adverse effects were detected following administration [23,24]. While preexisting anti-nanobody antibodies were found in one clinical study using a tetravalent agonistic nanobody targeting the Death Receptor 5 as an antitumor agent [25], no more preexisting anti-nanobody antibodies appear to be present than preexisting autoantibodies against conventional V_H_ fragments [26]. 

Nanobodies can be readily generated against many targets through the immunization of camelids with either the antigen of interest, DNA coding for it, or cells expressing the antigen on their surface. Subsequent amplification of the nanobody gene sequences from peripheral blood mononuclear cells yields an immune cDNA library from which specific nanobodies can be selected through various affinity (e.g., phage display) and/or functional screens [27]. Alternatively, synthetic libraries can be used. These generally utilize a fixed framework, where residues in the CDR regions are randomized to obtain specificity for different targets [28,29]. 

Nanobodies with single-digit nanomolar affinities should preferentially be selected for further *in vivo* applications, especially when the target is only expressed at low levels. The affinity of nanobodies across species furthermore facilitates their preclinical characterization, as the uptake in organs and tissues constitutively expressing the target can be better assessed. 

The selected nanobody clones can then straightforwardly be expressed in bacteria (e.g., *E. coli*) or yeast strains (e.g., *S. cerevisiae* and *P. pastoris)* at yields of several milligrams of soluble nanobodies per liter of culture. Their relatively simple and single gene form allows the engineering of nanobodies into all kinds of formats, including the generation of multimeric and multispecific compounds, creation of fusion proteins, and addition of small peptide sequences (tags) for later functionalization [30].

In the last 15 years, nanobodies have been proposed as a new class of antibody-derived agents for molecular imaging because of their unique features regarding affinity, specificity, and rapid pharmacokinetics, ensuring good uptake in the targeted tissues and high target-to-background ratios [31].

## 3. Radiolabeled Nanobodies for Same-Day, High-Contrast Nuclear Imaging and Targeted Radionuclide Therapy with Minimal Toxicity

### 3.1. Radiolabeling of Nanobodies

Nuclear molecular imaging requires the targeting moiety, in this case a nanobody, to be labeled with a diagnostic radioisotope. The latter can either be a gamma-emitting isotope for single photon emission computed tomography (SPECT) or a positron-emitting isotope for positron emission tomography (PET). Clinically, the higher resolution, sensitivity, and quantitative potential of PET/Computed Tomography (CT) imaging is driving its adoption and is expected to result in a shift towards the increasing use of this technology over SPECT/CT [32]. On the contrary, microSPECT devices specifically developed for small-animal imaging typically achieve higher spatial resolution than microPET systems. It is thus an attractive imaging technology for the initial in vivo screening and characterization of a set of nanobodies, especially since nanobodies can be easily labeled with ^99m^Tc. Conveniently, ^99m^Tc–tricarbonyl reacts site-specifically with a genetically inserted C-terminal hexahistidine tag, which can also be used for purification purposes via immobilized metal affinity chromatography [33]. 

Radiolabeling of proteins with other radiometals (e.g., ^64^Cu, ^68^Ga, and ^89^Zr for PET or ^67^Ga and ^111^In for SPECT) or radiohalogens (e.g., ^18^F and ^124^I for PET and ^123/131^I for SPECT) usually necessitates the use of a chelator for complexation or a prosthetic group for electrophilic substitution, respectively. For human applications, the PET isotopes ^68^Ga and ^18^F are particularly suited to imaging with nanobodies due to their short half-lives (68 and 110 min, respectively), which match up well with the nanobodies’ biological half-life. For the attachment of chelators and prosthetic groups to the nanobody, different conjugation strategies are possible. These can be broadly divided into two categories: random or site-specific labeling. Random labeling typically occurs through conjugation to primary amines (lysines) in the framework. Although this is a common and straightforward method, it however results in a heterogenous mixture with varying amounts of labels per nanobody at different positions. Contrarily, site-specific strategies aim to obtain homogenous and consistent tracers through the conjugation of a single contrast agent to predetermined, specific sites. Positioning of the contrast label opposite to the antigen-binding site furthermore avoids interference with the binding capacity of nanobodies [34]. Different types of site-specific labeling methods with nanobodies have been explored for in vivo applications. For example, the incorporation of a C-terminal cysteine tag enables reaction with maleimide-functionalized agents after the prior reduction of dimeric nanobodies or nanobodies with a blocked cysteine. Importantly, the reduction reaction must be carefully titrated to prevent disruption of the nanobodies’ internal disulfide bridges [35]. Although reversal of the thioether bond is known to occur in vivo [36,37], this is not expected to happen fast enough to pose a problem to nanobody probes due to their fast pharmacokinetics [34]. A more elegant method for site-specific conjugation is enzyme-mediated ligation through the transpeptidase Sortase A. Here, the enzyme catalyzes the formation of a new peptide bond between the peptide motif LPXTG expressed C-terminally on the nanobody and the label containing a N-terminal oligo-glycine motif [38,39]. Other methods under investigation for the design of site-specifically labeled nanobodies for molecular imaging are alkyne–azide click reactions and those involving the incorporation of unnatural amino acids into the nanobody structure [34,40]. 

### 3.2. In Vivo Biodistribution of Radiolabeled Nanobodies

Upon intravenous administration, radiolabeled nanobodies are rapidly cleared from the blood circulation. In mice, normally less than 0.5% Injected Activity (IA)/g remains present in the blood pool at 1 h post-injection [41,42,43,44]. In humans, the early- and late-phase half-life of the anti-human epidermal growth factor receptor 2 (HER2) ^68^Ga-labeled nanobody 2Rs15d was calculated as 2.9 and 25.5 min, respectively, and at 1 h post-injection of the tracer, only 10% of the injected activity remained in the blood pool [23]. The size of nanobodies being below 60 kDa causes them to be filtered through the glomeruli in the kidneys. However, nanobodies are subsequently reabsorbed by the proximal tubuli, resulting in their retention in the renal cortex. It has been previously shown that the endocytic receptor megalin, which is abundantly expressed in the brush border, is at least partially involved in the renal retention of nanobodies (megalin-deficient mice show 40% less renal retention of a ^99m^Tc-labeled nanobody than wild-type mice) [45]. The long-term renal retention of (radiolabeled) nanobodies and/or their (radio)catabolites can be an issue as it can possibly lead to undesired nephrotoxicity. Furthermore, the imaging of molecular targets in the vicinity of the kidneys is hampered due to intense renal signals. Therefore, several possible strategies to reduce the renal reabsorption of nanobodies have been investigated. Coadministration of positively charged amino acids or gelofusin, which competitively interact with megalin/cubulin receptors, have long been known to reduce the renal retention of radiometal-labeled antibody fragments and peptides [46,47], and this has also been confirmed for nanobodies [45,48]. Alternatively, the removal of charged amino-acid tags, for example, used for purification or radiolabeling purposes, has an effect on the polarity of nanobodies and consequently has an important impact on the degree of kidney retention. Indeed, Myc–His- and His-tagged nanobodies show considerably higher kidney values compared to their untagged analogues [48,49]. For clinical applications, the His tag is recommended to be removed anyway to prevent immunogenic reactions [50,51]. Finally, the degree of kidney retention for radiohalogenated (fluorinated and iodinated) nanobodies is significantly lower than for radiometal-labeled analogues. Catabolites of radiohalogenated compounds formed in the kidneys are thought to be nonresidualizing and hydrophobic and rapidly excreted via the urine [52,53,54]. 

Other than the accumulation in kidneys and urine, the uptake of radiolabeled nanobodies in nontargeted organs and tissues is very low (Figure 1). In combination with efficient penetration into and diffusion through tissues and fast targeting, this consequently results in high target-to-background ratios early after administration, allowing same-day imaging [55]. This is in stark contrast to full-length antibodies, where due to their long circulation time, optimal tumor-to-background contrast is only obtained several days after administration of the tracer and nonspecific uptake is generally much higher [11]. Other non-immunoglobulin low-molecular-weight protein scaffolds (e.g., Affibodies, DARPins, Adnectins, ADAPTs, or knottins) share many of the in vivo characteristics of nanobodies. A recent review discusses in detail the clinical application and promising preclinical developments of nanobodies and other small proteins for radionuclide-based imaging within the field of oncology [55]. The main advantage of nanobodies over these scaffolds remains the relatively simple process to generate new nanobodies with high affinity through the immunization of camelids. On the other hand, further size-reduced compounds could eventually be produced synthetically instead of via fermentation, once a lead compound has been identified [56]. 

### 3.3. Nuclear Medicine Applications with Nanobodies

A major application field for nanobody-based radiotracers is cancer imaging. Radiolabeled nanobodies targeting tumor biomarkers such as the epidermal growth factor receptor (EGFR), carcinoembryonic antigen (CEA), mesothelin, prostate-specific membrane antigen (PSMA), CD20, or HER2 showed high specific tumor uptake (ranging typically between 2 and 10% IA/g, depending on the expression level of the target) in preclinical tumor models as soon as 1 h after administration, with tumor-to-blood ratios of up to 10–30 (Figure 1B,C) [23,35,38,41,42,45,48,49,53,54,57,58,59,60,61,62,63,64,65,66,67,68,69,70,71]. Of particular interest is the anti-HER2 nanobody 2Rs15d that has been selected as a lead compound for clinical translation. In the very first clinical trial with a radiolabeled nanobody, Keyaerts et al. demonstrated that ^68^Ga–NOTA–2Rs15d PET/CT enabled the visualization of both primary lesions and/or local or distant metastases in HER2-positive breast cancer patients, without adverse effects (Figure 1H). Good tumor uptake was observed, with mean standard uptake values of up to 11.8 for primary tumors and 6.0 in metastases between 60 and 90 min post-injection. With the exception of the kidneys, intestines, and liver, background uptake was low (weak uptake in glandular tissues such as the salivary glands, pituitary, lacrimal glands, and axillary sweat glands was thought to be related to low levels of HER2 expression or chelator-mediated trapping mechanisms). Ultimately, 90 min post-injection was chosen as the optimal imaging time point, due to decreased liver uptake compared to 60 min post-injection. In this study, no preexisting or tracer-induced antibodies against the nanobody 2Rs15d could be detected. These findings imply the potential application of ^68^Ga–2Rs15d for the noninvasive assessment of the HER2 status of patients [23]. A phase II study with this tracer has since been initiated, evaluating its potential to detect brain metastasis in breast cancer patients (NCT03331601). 

Next to imaging the tumor cells themselves for the prognosis and prediction of therapy response, a different application is the characterization and quantification of specific immune cells within the tumor environment. This approach could eventually also aid in better understanding and evaluating drug action during drug development. In mice, radiolabeled nanobodies have been proven to be able to track the infiltration of CD11b (macrophages, dendritic cells, and neutrophils) and major histocompatibility complex (MHC) class II (macrophages and dendritic cells) positive cells in both xenogeneic and syngeneic tumors, as well as after injection of complete Freund’s adjuvant [72] (Figure 1D). Similarly, macrophage mannose receptor (MMR)-specific nanobodies were used to image tumor-associated macrophages in mice [44,74]. The human-MMR-specific nanobody MMR3.49 labelled with ^68^Ga is currently being translated to the clinic, with a phase I safety trial to be initiated soon [75]. Of growing interest is the prediction of immune-checkpoint blockade therapy outcome. Nuclear imaging with radiolabeled nanobodies against the T-cell marker CD8 or programmed death-ligand 1 (PD-L1) shows promising results as a tool to image antitumor immune responses [76,77,78,79]. 

Macrophage-specific nanobodies have also been found to be of interest for imaging in other inflammatory diseases, examples of which are hepatic inflammation and collagen-induced arthritis (CIA). Indeed, radiolabeled nanobodies targeting MMR or the complement receptor of the Ig superfamily (CRIg or VSIG4) were found to accumulate specifically in inflamed joints of mouse CIA models (Figure 1G). Furthermore, CRIg/VSIG4 imaging appeared to be sufficiently sensitive to detect early signs of inflammation, even before the manifestation of clinical signs [73,80,81,82]. 

Applications that heavily rely on an elevated uptake with low nonspecific surrounding background signals are the imaging of pancreatic islets after transplantation [83] and the imaging of vulnerable atherosclerotic plaques. The feasibility to noninvasively detect small, inflamed atherosclerotic lesions in the aortic arch of mice or along the aortas of atherosclerotic rabbits has been shown with radiolabeled nanobodies targeting MMR, vascular cell adhesion molecule-1 (VCAM-1) (Figure 1E), and lectin-like oxidized low-density lipoprotein receptor (LOX-1) [43,52,84,85,86,87,88,89]. However, their prognostic value for the identification of high-risk patients remains to be demonstrated in clinical trials. Within this context, the anti-VCAM-1 nanobody cAbVCAM1-5 is currently in the process of being translated into the clinic.

In analogy with molecular imaging, the incorporation of high-energy β^-^ (^177^Lu, ^131^I) or α-emitters (^211^At, ^213^Bi, and ^225^Ac) allows the use of nanobodies for targeted radionuclide therapy (TRNT). The nanobody is used as a vehicle to specifically bring damaging ionizing radiation to the tumor cells. Radiation can inflict damage to tumor cells either by direct DNA damage or by the generation of reactive oxygen species (ROS). Furthermore, the destruction of cancer cells can release antigens and immune-triggers into the environment, activating an anticancer immune response [90,91]. β-emitters, which have a lower linear energy transfer (LET), deposit their energy over a longer path length than α-emitters, and can thus be advantageous in heterogenous tumors where not all malignant cells express the molecular target. On the other hand, α-emitters deliver higher therapeutic absorbed doses, but might require internalization as these operate at very short distances. α-TRNT is mostly suggested for the treatment of micro-metastasis and minimal residual disease [92,93]. 

In a theranostic approach, therapy is combined with molecular imaging, the latter being used to predict susceptibility to TRNT and as a means to follow up treatment. This can be accomplished either by using the same nanobody for both the preparation of the diagnostic and therapeutic radio-analogue (e.g., pairing of ^68^Ga/^177^Lu or ^123/124^I/^131^I-labeled agents) or by radiolabeling the nanobody with a radioisotope with both diagnostic and therapeutic properties (e.g., ^177^Lu and ^131^I) [92,93]. This strategy has successfully been applied preclinically in breast and ovarian cancer, non-Hodgkin lymphoma, and multiple myeloma using anti-HER2 (Figure 1F), anti-CD20, and anti-idiotypic (multiple myeloma) nanobodies, respectively [49,54,68]. Efficient tumor therapy in a preclinical setting could be demonstrated through either significant improvement in overall survival compared to all controls in mice inoculated subcutaneously or intraperitoneally with HER2- or CD20-positive tumors cells, or the inhibition of disease progress in the 5T2 multiple myeloma mouse model. Furthermore, the fast pharmacokinetics and low nonspecific uptake of nanobodies lead to minimal toxicity. Contrarily, the prolonged blood residence time of monoclonal antibodies has important implications for toxicity to bone marrow and other highly perfused organs such as the spleen and liver. So far, renal toxicity due to kidney retention has not been observed in mice, even after repeated administration. Furthermore, by using ^131^I-labeled nanobodies and having nonresidual catabolites in the kidneys, the absorbed dose to the kidneys could even be reduced below the dose delivered to the tumor [49,54,68]. A phase I trial using a low-dose ^131^I-labeled anti-HER2 nanobody 2Rs15d in breast cancer patients has recently been completed (NCT02683083) [94].

While monovalent nanobodies are most often used for nuclear imaging and TRNT, the use of multimeric nanobodies has also been investigated. Dimeric monospecific nanobodies generally show similar pharmacokinetics to monovalent tracers with regard to fast blood elimination and renal clearance. Tumor uptake of bivalent nanobodies at early time points has been found to be slightly lower than that of monovalent ones, although the bivalent compound shows considerably longer tumor retention [69]. However, due to the increased avidity and size of bivalent nanobodies, they likely exhibit inferior tumor penetration properties and are more limited to the perivascular region [95,96]. Bivalent nanobodies can consequently be used as a means to cope with on-target off-tumor uptake. This was demonstrated by Movahedi et al., who used a bivalent unlabeled anti-MMR nanobody to modify the biodistribution of the radiolabeled monomeric nanobody. Impact on tumor uptake was minimal, while specific uptake in nontumor organs and tissues was almost completely blocked [44]. When extension of the blood half-life is desirable, multimeric constructs containing one or more tumor-specific domains and an albumin-binding domain can be engineered. The resulting radiolabeled compounds show vastly increased blood retention, and consequently, tumor values significantly increase over time, although so does the nonspecific uptake [69,97,98,99]. Half-life-extended nanobodies are thus theoretically more relevant in a therapeutic context, and less so for diagnostic applications. The negative impact on deep tumor penetration should also be considered. 

An overview of nanobody-based radiopharmaceuticals with potential application in clinical nuclear medicine is provided in Table 1.

## 4. Image-Guided Surgery and Photodynamic Therapy Using Fluorescent Nanobodies

### 4.1. Design of Fluorescent Nanobody-Based Tracers

Alternatively to nuclear imaging, fluorescence imaging requires the presence of a fluorescent label for sensitive detection. While a wide range of fluorescent dyes are available for biotechnological purposes, for in vivo imaging applications, the choice of fluorophore is limited to those emitting in the near-infrared (NIR) region, and more specifically, those with a maximal excitation and emission wavelength between 650 and 900 nm. In this range, scattering, nonspecific tissue autofluorescence and absorption by endogenous chromophores are minimal. These characteristics infer intrinsically improved signal-to-background ratios and as such provide sharper contrast, better resolution, and deeper signal detection (from several mm to 1 cm) [107]. Commonly, hydrophilic (sulfonated) variants of cyanine dyes with a penta- or heptamethine chain are used, e.g., Cy5, Alexa Fluor (AF)680, IRDye680RD, or IRDye800CW. The actual choice of the dye will depend on the specifications of the camera system used for detection (the wavelength(s) the system is able to detect) and whether the tracer is intended to be used in combination with a second tracer providing complementary information, e.g., highlighting of the tumor tissue to be resected and the nerves to be preserved (multiplexing). 

**Figure 2:**
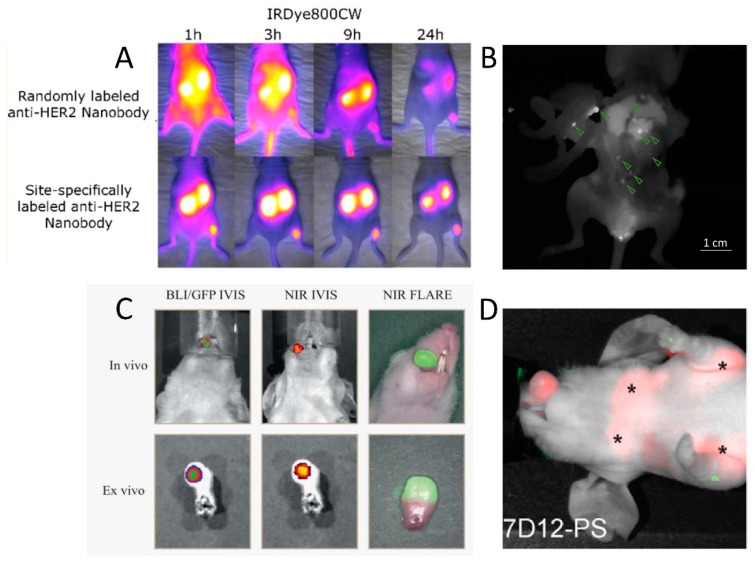
Examples of in vivo fluorescent molecular imaging with nanobody tracers in mouse tumor models. (**A**) Comparison of the biodistribution and tumor-targeting potential of the anti-HER2 nanobody 2Rs15d conjugated with IRDye800CW either randomly (top) or site-specifically (bottom). Adapted with permission from [111]. Copyright 2017 American Chemical Society. (**B**) Fluorescence image acquired during the surgical resection of intraperitoneally disseminated HER2+ tumor lesions. Site-specifically IRDye800CW-labeled 2Rs15d nanobody was injected 90 min before surgery. Fluorescent signal in tumor lesions (indicated by green arrows) is clearly discernible from background signal. Adapted with permission from [123]. (**C**) Real-time fluorescence imaging of orthotopic tongue tumor 24 h post-injection of an EGFR-specific randomly IRDye800CW-conjugated nanobody. Colocalization with bioluminescence imaging (BLI) and green fluorescent protein (GFP) signals is shown. Adapted with permission from [109]. (**D**) Fluorescent imaging of an orthotopically inoculated tongue tumor at 1 h post-injection of an EGFR-specific randomly IRDye700DX-conjugated nanobody for photodynamic therapy (PDT). Stars denote the presence of fluorescent tracer uptake in invaded lymph nodes. Adapted with permission from [125].

Analogous conjugation strategies to those described above for chelators and prosthetic groups are applicable for fluorophore conjugation. However, it is increasingly being recognized that fluorophore conjugation can have a significant impact on the pharmacokinetics of antibody tracers [108]. The impact is most likely even more pronounced for smaller fragments such as nanobodies. In fact, randomly IRDye800CW-labeled nanobodies have been demonstrated to have an atypical tissue distribution with high background signals, high liver accumulation, and low tumor contrast [109,110,111]. Likewise, randomly IRDye680RD- and AF680-labeled nanobodies do not exhibit such persistent background signals, but are partially excreted via the hepatobiliary route [111,112,113]. The chosen conjugation chemistry appears to be an additional determining factor, as the site-specific labeling of IRDye800CW and IRDye680RD via a C-terminal cysteine tag yields nanobody tracers with normal biodistribution profiles, meaning fast tumor targeting, renal excretion, and no nonspecific uptake (Figure 2A) [111]. Conjugation of more than one dye per nanobody is furthermore undesirable since a higher dye/nanobody ratio may cause quenching of the fluorescent signal due to the close proximity of the dyes.

Compared to nuclear imaging techniques, fluorescent imaging requires sufficient uptake of the tracer in the tissue of interest for sensitive detection. Because of the depth-related attenuation of fluorescent signals, high injected doses are often required in humans, at the limits of or above the microdose level (less than 30 nmol or 100 µg [114]) [115,116,117,118,119]. Moreover, as for equal doses, the maximal tumor signal that can be attained will be lower for nanobodies than for long-circulating antibodies, a higher molar concentration will likely be needed as well [113]. In mice, typically, 1–5 nmol of fluorescent nanobody based on the dye concentration (25–75 µg of protein) is injected. Adjusting the injected dose does not appear to significantly affect the biodistribution of nanobodies (in comparison, a higher dose of antibodies results in an increase of nonspecific signal), and dose optimization could thus lead to superior image quality in terms of signal intensity and tumor-to-background ratios [113]. The use of radio- or bimodal (radioactivity in combination with fluorescence)-labeled tracers could be an alternative approach to increase sensitivity for the intraoperative detection of deeper located lesions, but this remains to be investigated for nanobodies [120].

### 4.2. Fluorescence-Guided Surgery Using Nanobody-Based Contrast Agents

Several fluorescently labeled nanobodies have been successfully evaluated in the context of intraoperative imaging (an overview is provided in Table 2). IRDye800CW-labeled anti-EGFR nanobodies could clearly delineate orthotopic tongue tumors in mice, and even enabled the identification of a lymph node metastasis (Figure 2C) [109]. Of note, in this study the optimal imaging time point appeared to be 24 h post-injection, likely due to the random characteristic of the labeling method. The site-specifically IRDye800CW- and IRDye680RD-labeled nanobodies 11A4 and B9, respectively targeting HER2 and carbonic anhydrase IX (CAIX), showed accumulation in breast cancer lesions (DCIS) and lung metastasis in an experimental setup mimicking the surgical setting [121,122]. Furthermore, it was demonstrated that the combination of nanobodies targeting these two independent tumor markers, but labeled with the same fluorescent dye, could further improve tumor-to-background ratios and overcome tumor heterogeneity [122]. Finally, using a mouse model of intraperitoneal disseminated tumor lesions mimicking late-stage ovarian cancer, the advantage of fluorescence guidance with the anti-HER2 nanobody tracer 2Rs15d-IRDye800CW on the efficiency of debulking surgery was demonstrated. Submillimeter lesions could be visualized with high contrast at 1.5 h post-injection, leading to the excision of significantly more tumor tissue as compared to traditional surgery and resection of less false-positive tissue (Figure 2B) [123]. 

Similarly to the theranostic approach in nuclear medicine, where TRNT is combined with diagnostic imaging, the conjugation of a photosensitizer to a tumor-targeting nanobody enables its use for image-guided resection followed by photodynamic therapy (PDT) of residual malignant cells. In PDT, a photosensitizer is activated by incidence light to produce ROS. These ROS can damage the tumor by directly causing cell death through apoptosis and necrosis, damaging the tumor vasculature, and inducing an immune response [126]. This was investigated in a mouse orthotopic tongue tumor model with EGFR-specific nanobodies conjugated randomly to the photosensitizer IRDye700DX (Figure 2D). The effectiveness of a monovalent nanobody and bispecific variant, which binds two different sites on EGFR, was compared with that of a conventional antibody. Both nanobody photosensitizers outperformed the antibody after therapeutic illumination, with more homogenous damage to the tumor and less nontarget damage. Furthermore, despite the higher internalization seen in vitro for the bispecific variant, better in vivo therapeutic results were obtained with the monovalent nanobody. This is in accordance with the assumption that smaller, monovalent compounds diffuse more homogenously through tumor tissue [125,127].

## 5. Conclusions and Perspectives

Nanobodies, with their unique properties, show great promise as targeting moieties in molecular imaging and therapy. Their fast blood clearance, rapid and homogenous tissue penetration, and low background retention allow highly specific imaging at early time points after administration and effective therapy with minimal nonspecific toxicity. The utility of nanobody tracers is now broadly recognized thanks to the convincing preclinical data obtained so far. However, clinical data on their use in this field is still very limited. The expensive and time-consuming process required to translate nanobodies into the clinic (current Good Manufacturing Practice (cGMP) production, toxicity studies, and Investigational Medicinal Product Dossier (IMPD) filing) is probably the major limiting factor. This, however, holds true for any molecular tracer. 

The first clinically translated radiolabeled nanobody, the anti-HER2 nanobody 2Rs15d, labeled either with ^68^Ga or ^131^I, has been investigated in two phase I trials as a potential tool to provide predictive and responsive information on targeted tumor therapies. Follow-up studies respectively evaluating the diagnosis and treatment of breast cancer brain metastasis are now ongoing or planned. In the next years, clinical data of two additional radiolabeled nanobodies is expected, as their clinical translation is almost completed. A nanobody targeting the inflammatory marker VCAM-1 will be evaluated for vulnerable plaque screening, and a macrophage-specific nanobody will also be investigated, which opens up opportunities to image immune cell activation and dynamics in oncology and inflammatory diseases. The latter approach is expected to be further exploited in the future to aid in the development, selection, and monitoring of (novel) immunotherapies. 

In the context of intraoperative imaging, properly designed fluorescent nanobody tracers seem to be promising tools to assist and guide surgeons during complex interventions. Evaluated only in a preclinical setting so far, their feasibility and surgical benefit in humans remains to be demonstrated. The strategy to move most rapidly towards the clinic would be to fluorescently label clinical-grade nanobodies which are already available (e.g., anti-HER2 nanobody), in analogy with antibodies currently under investigation as fluorescent contrast agents. However, preferentially, novel nanobodies targeting more relevant biomarkers for the application of image-guided surgery are expected to be developed and clinically translated.

Regarding the design of nanobody tracers, further advances towards novel chemistries permitting conjugation of contrast labels in a more controlled manner are warranted, as ultimately, any labeling method that is considered for clinical translation must be evaluated in a regulatory context. Furthermore, developments of chelators, prosthetic groups, fluorescent dyes, and bimodal labels with improved effects on the pharmacokinetics of nanobodies would be of interest, especially related to kidney retention. This aspect remains a critical point for potential toxicity issues, particularly for therapeutic applications.

## Figures and Tables

**Table 1:** Overview of preclinically and clinically tested nanobody-based radiopharmaceuticals with applications in nuclear medicine.

Application Field	Molecular Target	Lead Compound	Radiolabel	Disease	Development Phase	References
Tumor cell imaging/therapy	HER2	2Rs15d	^99m^Tc, ^111^In, ^177^Lu, ^18^F, ^225^Ac	Breast cancer	Preclinical	[42,57,58,59,60,100]
			^68^Ga		Phase II ongoing (NCT03331601)	[23,41]
			^131^I		Phase I completed (NCT02683083)	[54,94]
		5F7	^125^I, ^131^I, ^18^F		Preclinical	[60,61,62,63]
	EGFR	7C12,7D12	^99m^Tc, ^177^Lu, ^68^Ga, ^89^Zr	Skin cancer	Preclinical	[45,64,65,97]
		D10	^99m^Tc			[66]
	HER3	MSB0010853	^89^Zr	Non-small cell lung cancer, head and neck cancer	Preclinical	[99]
	PSMA	PSMA30	^99m^Tc	Prostate cancer	Preclinical	[67]
		JVZ-007	^111^In			[48]
	CEA	CEA5	^99m^Tc	Colon cancer	Preclinical	[71]
	Mesothelin	A1	^99m^Tc	Breast cancer	Preclinical	[70]
	CD20	9077, 9079	^99m^Tc, ^111^In, ^177^Lu, ^68^Ga	Non-Hodgkin lymphoma	Preclinical	[68,69]
	HGF	1E6-Alb8, 6E10-Alb8	^89^Zr	Glioma	Preclinical	[98]
	Mouse monoclonal protein	R3b23	^99m^Tc, ^177^Lu	Multiple myeloma	Preclinical	[101]
Tumor immunology and inflammatory diseases	Mouse CD8	VHH-X118	^89^Zr	Tumor immunology	Preclinical	[76]
	Mouse PD-L1	B3	^18^F	Immune checkpoint	Preclinical	[78]
		C3,E2	^99m^Tc			[77]
	Mouse dendritic cells	DC1.8, DC2.1	^99m^Tc	Tumor immunology	Preclinical	[102]
	Mouse Cd11b	VHHDC13	^18^F, ^64^Cu	Tumor immunology	Preclinical	[72]
	Mouse MHC class II	VHH7	^18^F, ^64^Cu	Tumor immunology	Preclinical	[39,72]
	Human MHC class II	VHH4	^64^Cu	Graft vs. host disease	Preclinical	[103]
	Mouse MMR	MMRCl1	^99m^Tc	Tumor immunology	Preclinical	[44]
			^99m^Tc	Arthritis	Preclinical	[81]
		MMR3.49	^99m^Tc, ^18^F, ^68^Ga	Tumor immunology	Clinical translation	[74,75]
	Human MMR	MMR3.49	^99m^Tc, ^64^Cu, ^68^Ga	Atherosclerosis	Preclinical	[84,86]
	CRIg/VSIG4	VM119	^99m^Tc, ^18^F	Arthritis, liver inflammation	Preclinical	[73,82]
	Clec4F	C4m22	^99m^Tc	Liver inflammation	Preclinical	[82]
	VCAM-1	cAbVCAM1-5	^99m^Tc, ^111^In, ^18^F, ^64^Cu, ^68^Ga	Atherosclerosis	Clinical translation	[43,52,85,86,87,89]
	LOX-1	Lox1.14	^99m^Tc, ^64^Cu	Atherosclerosis	Preclinical	[86,88]
Amyloidosis	Gelsolin	FAF Nb1	^99m^Tc	Gelsolin amyloidosis	Preclinical	[104,105]
	B-amyloid	Ni3A, pa2H	^99m^Tc	Alzheimer’s	Preclinical	[106]
Diabetes	DPP6	4hD29	^99m^Tc, ^111^In	Diabetes	Preclinical	[83]

HER2: human epidermal growth factor receptor 2, EGFR: epidermal growth factor receptor, HER3: human epidermal growth factor receptor 3, PSMA: prostate-specific membrane antigen, CEA: carcinoembryonic antigen, HGF: hepatocyte growth factor, PD-L1: programmed death-ligand 1, MMR: macrophage mannose receptor, CRIg/VSIG4: complement receptor of the immunoglobulin family/V-set and immunoglobulin domain containing 4, Clec4F: C-type lectin domain family 4 member F, VCAM-1: vascular cell adhesion molecule 1, LOX-1: lectin-like oxidized low-density lipoprotein receptor-1, DPP6: dipeptidyl peptidase like 6.

**Table 2:** Overview of in vivo preclinically evaluated fluorescent nanobodies with potential for clinical interventional molecular imaging and photodynamic therapy.

Molecular Target	Lead Compound	Fluorophore	Conjugation Strategy	Intended Clinical Application	References
HER2	2Rs15d	IRDye800CW IRDye680RD	Random (Lys–NHS)	-	[111]
		IRDye800CW IRDye680RD	Site-specific (Cys–maleimide)	Intraoperative imaging of breast/ovarian cancer	[111,123]
		Cy5	Site-specific (Sortase A)	Intraoperative imaging of breast cancer	[38]
	11A4	IRDye800CW IRDye680RD	Site-specific (Cys–maleimide)	Intraoperative imaging of breast cancer	[122,124]
CAIX	B9	IRDye800CW	Site-specific (Cys–maleimide)	Intraoperative imaging of breast cancer	[121,122]
EGFR	7D12	IRDye800CW	Random (Lys–NHS)	Intraoperative imaging of head and neck cancer	[109]
	7D12, 7D12-9G6	IRDye700DX	Random (Lys–NHS)	Photodynamic therapy of head and neck cancer	[125]

NHS: N-Hydroxysuccinimide, HER2: human epidermal growth factor receptor 2, CAIX: carbonic anhydrase 9, EGFR: epidermal growth factor receptor.
